# Mouse testicular macrophages can independently produce testosterone and are regulated by Cebpb

**DOI:** 10.1186/s40659-024-00544-8

**Published:** 2024-09-09

**Authors:** Nengliang Duan, Yuanshuai Ran, Huapei Wang, Ya Luo, Zhixiang Gao, Xingyu Lu, Fengmei Cui, Qiu Chen, Boxin Xue, Xiaolong Liu

**Affiliations:** 1 Department of Urology, The Second Affiliated Hospital of Soochow University, NO.1055 SanXiang Road, Suzhou, Jiangsu Province 215000 China; 2 State Key Laboratory of Radiation Medicine and Protection, School of Radiation Medicine and Protection, Medical College of Soochow University, Suzhou, China

**Keywords:** Testicular macrophage, Testosterone, De novo synthesis, Cebpb

## Abstract

**Background:**

Testicular macrophages (TM) have long been recognized for their role in immune response within the testicular environment. However, their involvement in steroid hormone synthesis, particularly testosterone, has not been fully elucidated. This study aims to explore the capability of TM to synthesize and secrete testosterone de novo and to investigate the regulatory mechanisms involved.

**Results:**

Transcriptomic analysis revealed significant expression of *Cyp11a1*, *Cyp17a1*, *Hsd3b1*, and *Hsd17b3* in TM, which are key enzymes in the testosterone synthesis pathway. qPCR analysis and immunofluorescence validation confirmed the autonomous capability of TM to synthesize testosterone. Ablation of TM in mice resulted in decreased physiological testosterone levels, underscoring the significance of TM in maintaining testicular testosterone levels. Additionally, the study also demonstrated that Cebpb regulates the expression of these crucial genes, thereby modulating testosterone synthesis.

**Conclusions:**

This research establishes that TM possess the autonomous capacity to synthesize and secrete testosterone, contributing significantly to testicular testosterone levels. The transcription factor Cebpb plays a crucial role in this process by regulating the expression of key genes involved in testosterone synthesis.

**Supplementary Information:**

The online version contains supplementary material available at 10.1186/s40659-024-00544-8.

## Background

Testosterone is the primary sex steroid hormone and anabolic hormone in the male body, playing a crucial role in male sexual development, masculinization, and sperm production [1]. Additionally, it has significant effects on health for both males and females, including the regulation of libido, strength, immune function, hematopoietic function, and resistance to osteoporosis [2–5]. In the male body, it is known that, aside from a very small portion synthesized by the adrenal cortex zona reticularis, the majority of testosterone is synthesized by the Leydig cells within the testis [6]. During the developmental, two distinct populations of murine Leydig cells emerge sequentially. Fetal Leydig cells (FLC) appear shortly after the formation of testicular organs, producing androstenedione; subsequently, this androstenedione is converted into testosterone under the influence of Sertoli cells expressing Hsd17b3. Adult Leydig cells (ALC) arise in the pre-pubertal phase, possessing the capability to express Hsd17b3 and independently secrete testosterone [7]. After synthesis, testosterone is secreted into the bloodstream, maintaining male secondary sexual characteristics and functions. It also plays a vital role in promoting the development of germ cells into sperm and influencing the body’s synthetic metabolism. Disruption in testosterone synthesis can lead to a series of adverse consequences.

Research to date has indicated that the majority of white blood cells express steroid receptors, suggesting the possibility of direct regulation by steroids. Moreover, immune cells are not merely passive targets of steroid signal transduction; they represent a cell lineage closely associated with steroids. It has been reported that human alveolar macrophages, guinea pig alveolar macrophages, and human synovial macrophages actively participate in the metabolism and synthesis of sex steroid hormones [8–10]. Local steroid production in other tissues, such as the nervous system, has also been documented [11]. Interestingly, immune-related tissues also exhibit enzyme activity for steroid metabolism, with the intestine and lungs converting precursors into glucocorticoids during type 2 immune activation [12, 13].

In the testis, the primary immune cell population is macrophages, and these macrophages are closely linked to the function of Leydig cells. TM can assist testosterone production by generating 25-hydroxycholesterol, serving as a substrate for Leydig cells [14–17]. Interestingly, macrophages themselves also express the transcription factors required for steroid synthesis and can directly produce pregnenolone. This type of steroid may contribute to a local feedback loop between interstitial cells and macrophages, thereby regulating the production of testosterone [18]. Some studies also suggest that TM can produce a significant amount of corticosterone in vitro, although it remains unexplored whether this corticosterone is derived from the conversion of other steroids or synthesized de novo [19]. Therefore, it is essential to investigate whether TM possess steroidogenic capabilities and to what extent testicular function relies on the generation of these steroids.In this study, we propose that TM actively participate in the synthesis and secretion of testosterone de novo. Simultaneously, we have identified that this process within macrophages is regulated by the transcription factor Cebpb. The research provides a detailed demonstration of the scientific validity and importance of macrophages synthesis and secretion of testosterone. This is crucial for a comprehensive understanding of the intricate connection between immune regulation and sex steroid metabolism in the body.

## Materials and methods

### Animals

The mice used in the study were provided by Beijing Vital River Laboratory Animal Technology Co., Ltd. (Beijing, China). All animals were housed in SPF-grade facilities at the Animal Experiment Management Center of Soochow University. The procurement and use of these experimental animals followed the guidelines for animal care at Soochow University, adhering to the standards of experimental animal management. Before euthanasia, approximately 0.4 ml of blood was collected from each mouse via cardiac puncture. The blood was then centrifuged at 4000 g for 15 min at 4 °C to collect the serum, which was subsequently stored at -80 °C.

### Mouse testicular interstitial fluid (TIF) collection

TIF samples were bilaterally collected from the testis of each mouse following a method reported by Li X et al [20]. Briefly, the testes were immediately weighed and decapsulated after removal. They were then placed in HBSS in a proportion of 10 µL per milligram of testicular weight and shaken at 120 rpm for 5 min at room temperature to allow the interstitial fluid to be fully diluted in the HBSS solution. The suspension was then centrifuged at 300× g for 5 min at 4 °C, and the supernatant containing the TIF was stored at -80℃ before testosterone hormone testing. The tissue collected in the sediment was used for subsequent experiments.

### Mouse testicular interstitial cell isolation

The tissue sediment from the previous step was immersed in 600 µL HBSS solution containing 0.25 mg/ml Type IV collagenase (Sigma) and shaken at 120 rpm at 37 °C for 15 min to dissociate interstitial cells from the seminiferous tubules. The suspension was passed through a 40 μm filter to remove seminiferous tubules and large cell clumps. The cell suspension was then centrifuged, and the sediment represented the interstitial cells. After washing twice with HBSS, the cells were prepared for subsequent experiments.

### Isolation of primary TM

F4/80-positive macrophages were purified from the isolated testicular interstitial cells using MicroBeads conjugated to monoclonal anti-mouse F4/80 antibodies (Miltenyi Biotech, 130-110-443). The testicular single-cell suspension was magnetically labeled according to the protocol provided by Miltenyi Biotech to positively select intact cells expressing F4/80 protein. All cells were counted under a microscope.

### Preparation of whole testis single cell suspension

The enzyme solution was prepared using HBSS, which includes 1 mg/mL Type IV collagenase (Sigma), 1 mg/mL DNase I (Sigma), and 1 mg/mL hyaluronidase (Sigma). After the removal of the tunica albuginea from the mouse testis, the tissues were minced into small pieces. Each testis was then digested with 3 mL of the enzyme solution at 37 °C. The specific steps were conducted according to the protocol provided by the GentleMACS Dissociator (Miltenyi Biotech): the solution containing tissue and enzymes was transferred to a GentleMACS C tube and placed in the Dissociator. Three consecutive mechanical dissociations were performed, followed by the collection of the cell suspension.

### RNA extraction and gene expression analysis

Ribosomal RNA was removed from the samples using the NEBNext rRNA Depletion Kit (New England Biolabs, Massachusetts, USA). The NEBNext Ultra II Directional RNA Library Prep Kit (New England Biolabs, Massachusetts, USA) was employed for sequencing library construction. Quality control and quantification of the libraries were performed using the BioAnalyzer 2100 system (Agilent Technologies, USA), followed by 150-bp paired-end sequencing on the Illumina NovaSeq platform.

### RNA sequencing

The raw data were obtained using the Illumina NovaSeq 6000 sequencing platform. Initially, quality control of the raw data was performed using Q30 values. Cutadapt (v1.9.3) was utilized to remove adapters and low-quality reads, generating high-quality clean reads [21]. The clean reads were aligned to the reference genome using hisat2 (v2.0.4) [22]. Subsequently, guided by the Ensembl GTF gene annotation file, cuffdiff software was employed to obtain the mRNA expression profile [23, 24].

### Estimation of cell fractions

To conduct external validation, we gathered publicly annotated single-cell sequencing data from GSE112393 and used it as reference data. We utilized CIBERSORTx to estimate the relative cellular fractions of various testicular cells. This method is aimed at quantifying the relative proportions of different cell types in complex gene expression mixtures and has been extensively used for various diseases [25]. The single-cell expression matrix data were inputted into the CIBERSORTx website to compute Signature Genes. Subsequently, the data to be analyzed were inputted, and the relative cell proportions were calculated based on the reference expression characteristics of the genes.

### Single-cell sequencing analysis and gene set correlation analysis

We selected sequencing data from interstitial cells in the testis from GSE112393 for dimensionality reduction and clustering. We defined *Cyp11a1*, *Cyp17a1*, *Hsd3b1*, and *Hsd17b3* as the testosterone synthesis gene set, and *Apoe*,* Adgre1*,* Cd68*,* Lyz2*, and *C1qa* as the macrophage marker gene set. Utilizing the scMetabolism R package, we scored the expression of these two gene sets in each cell. We then performed a correlation analysis between the two gene sets. The results were displayed as scatter plots using the ggplot2 R package.

### Flow cytometry, fluorescence-activated cell sorting (FACS) and cell culture

For cells necessitating identification and sorting, surface staining was immediately conducted post-acquisition using F4/80 antibody (Biolegend, 123120, 1:50), Cd45 (Ebioscience, 15045182, 1:50), Cd11b (Biolegend, 101208, 1:100), Cd68 (Biolegend, 137013, 1:100) and Ctrl Antibody (Biolegend, 400525, 1:50). This was followed by a 30-minute incubation in darkness. Subsequently, the cells underwent three PBS washes, were resuspended, and then tested and sorted on the SH800S automatic flow cell sorter (Sony, Japan). Data collected from this process were meticulously analyzed using FlowJo software. The sorted cells were used for RNA extraction and in vitro cell culture. Cells were cultured in DMEM/F12 medium (WISENT, 319 − 085) containing 100 units/mL of penicillin and 100 µg/mL of streptomycin (WISENT,450 − 201), 5% horse serum (Pricellar, 164215–100), and 2.5% fetal bovine serum (Muticell, 085–150), and placed in an incubator with 5% CO2.

### RNA extraction and quantitative real-time PCR

Cells from both the directly collected samples and the cultured cells were collected and washed with PBS. Total RNA was extracted using the Total RNA Isolation kit (Vazyme Biotech, China). Reverse transcription was performed using HiScript III RT SuperMix (+ gDNA wiper) (Vazyme Biotech) according to the manufacturer’s instructions, utilizing 1 µg of RNA for cDNA synthesis. Quantitative PCR (qPCR) was conducted using Taq Pro Universal SYBR qPCR Master Mix (Vazyme Biotech) for fluorescence quantification. Target gene expression levels were normalized to Rn18s as a reference gene. The primer sequences are shown in Table 1.

**Table 1:** Counts values of partial genes in 3 testicular macropage samples

Gene	Forward primer nucleotide sequence (5′→3′)	Reverse primer nucleotide sequence (5′→3′)
*Pecam1*	CCAAAGCCAGTAGCATCATGGTC	GGATGGTGAAGTTGGCTACAGG
*Cd3e*	GCTCCAGGATTTCTCGGAAGTC	ATGGCTACTGCTGTCAGGTCCA
*Mc2r*	CCACAGTGCTCACCTTCACATC	TAGCATGGGAGCGGGCAAGTAA
*Nr2f2*	CGCCGAGTATAGCTGCCTCAAG	CTGGCTCCTAACGTACTCTTCC
*Ddx4*	GGACGAGATTTGATGGCTTGTGC	AGCGACTGGCAGTTATTCCATCC
*Sult1e1*	CTTCCAGGAGATGAAGAACAATCC	GGAAGTGGTTCTTCCAGTCTCC
*Cd68*	GGCGGTGGAATACAATGTGTCC	AGCAGGTCAAGGTGAACAGCTG
*Rn18s*	AGGCGCGCAAATTACCCAATCC	GCCCTCCAATTGTTCCTCGTTAAG
*Hsd17b3*	AAGACCGCCGATGAGTTTGT	TGCTGATGTTGCGTTTGAGG
*Cyp17a1*	GCCCAAGTCAAAGACACCTAAT	GTACCCAGGCGAAGAGAATAGA
*Hsd3b1*	GTATTCCGACCAGAAACCAAGG	GGCACACTTGCTTGAACACAG
*Cyp11a1*	CGCTTTTCCTTTGAGTCCATC	TCTGGAGGCAGGTTGAGCAT
*Lhcgr*	AATGGGACGACGCTAATCTCGC	TGAGCGTCTGAATGGACTCCAG
*Cyp11a1-CR*	TAGCAGCGTTATTCTGTAGGCA	CACAACTGGTACTGTAAACCAAATC
*Hsd3b1-CR*	TCTCCTTGATTCTCAGAATTCTTG	TCAGGGGCAGCTTCAAGGAT
*Cyp17a1-CR*	GAGAGATGGCTCAAATGTAATAAAT	CAGGTAAAACACTCATGCCTATAAA
*Hsd17b3-CR*	GTCTTCCTACTTCCACTTCTCG	CTCACTTTTAGACCATACTTTCCAG

### Agarose gel electrophoresis

Agarose powder was mixed with 1X TAE buffer and a one ten-thousandth concentration of GelRed to prepare a 3% agarose gel. DNA samples were mixed with 6× loading buffer (Takara). Five microliters of each sample were loaded into the wells. Electrophoresis was conducted at 80–100 V. DNA bands were observed and photographed under ultraviolet light.

### Immunofluorescence

Primary macrophages were seeded in a 96-well plate with round coverslip. After 24 h, they were washed with PBS, and the adherent cells were fixed with 4% paraformaldehyde (PFA) for 15 min. Subsequently, they were incubated with anhydrous methanol on ice for 10 min. Prior to applying the primary antibody, the non-specific background was blocked with PBS containing 5% BSA. The primary antibody included: rabbit antibodies against Cyp11a1 (abcam, ab175408, 1:100), Cyp17a1 (proteintech, 14447-1-AP, 1:100), Hsd3b1 (abclonal, a8035, 1:100), Hsd17b3 (proteintech, 13415-1-AP, 1:100), Lhcgr (proteintech, 26424-1-AP, 1:100), Testosterone (Testo) (Biomatik, CAU27861, 1:100) and Rat antibody against F4/80 (BIORAD, MCA497G). The cells were incubated with the primary antibodies overnight at 4 °C. Alexa Fluor 488 conjugated anti-rabbit IgG (Jackson ImmunoResearch, 705-545-147) and Cy3 conjugated anti-rat IgG (Jackson ImmunoResearch, 112-166-143) were used as secondary antibodies. After incubating at room temperature for 2 h, DAPI (Sigma) was used for nuclear staining. After mounting with fluorescent mounting media, laser confocal microscope was used for observation.

### Testosterone hormone analysis

The culture medium harvested from cell cultures, along with a control blank medium, collected mouse serum, and testicular interstitial fluid dissolved in HBSS, were all subjected to testosterone hormone analysis using chemiluminescence methods or targeted steroid hormone metabolomics techniques. The hormone concentration in the mouse testicular interstitial fluid was determined based on the concentration after dilution in HBSS solution.

### Construction of testicular macrophage-depleted mouse model

To deplete macrophages in the testis of mice, adult wild-type C57BL/6J mice were treated with rat-derived InVivoMAb anti-mouse CSF1, following the methods described in the literature with slight modifications [26]. A total of 12 mice were randomly assigned to the depletion group (*n* = 6) and the control group (*n* = 6). Each mouse was injected with 200 µg of InVivoMAb anti-mouse CSF1 (Bio X Cell, clone 5A1) or InVivoMAb rat IgG1 isotype control (Bio X Cell, clone HRPN) every other day. Samples were collected after four consecutive treatments.

### Tissue preparation and immunohistochemistry

Testis samples were fixed in modified Davidson’s solution or 4% formaldehyde solution and fixed overnight at room temperature [27]. After trimming and dehydration, the sample was embedded in paraffin and prepared into 4 μm sections for immunohistochemical staining. Slices were dewaxed in dimethylbenzene and treated with a series of decreasing concentrations of ethanol, followed by boiling in antigen retrieval solution for 100 s. The cooled slices were incubated with 3% H2O_2_ at room temperature for 15 min and blocked with 5% bovine serum for 1 h. The following antibodies were used for immunohistochemistry: rabbit antibodies against Sox9 (abcam, ab185966, 1:3000), Cyp17a1 (proteintech, 14447-1-AP, 1:150), Plzf (abclonal, A5863, 1:100) and Testosterone (Testo) (Biomatik, CAU27861, 1:100). The sections were incubated overnight at 4 °C. GTVisionTM III Detection System/Mo&Rb (Including DAB) Immunoassay Kit (Genetech, GK5007, China) is used for staining.

### Prediction of transcription factor

The NCBI database (https://www.ncbi.nlm.nih.gov/) contains genomic sequence information for all genes. In this study, we retrieved the promoter region sequences of the four genes *Cyp11a1*, *Cyp17a1*, *Hsd3b1*, and *Hsd17b3*. By analyzing these promoter regions, we identified potential transcription factors and their binding sites that may be associated with the regulation of these genes’ expression. The PROMO database (https://alggen.lsi.upc.es/cgi-bin/promo_v3/promo/promoinit.cgi?dirDB=TF_8.3) was used to identify the putative binding sites of transcription factors in DNA sequences, assisting in predicting potential transcription factors [28, 29].

### Transient transfection

Isolated primary TM were cultured in vitro for 2 h until the cells adhered to the surface. Subsequently, the culture medium was replaced, and siRNA targeting *Cebpb* was introduced into the cells using RNAiMAX transfection reagent (Invitrogen). Simultaneously, a plasmid overexpressing *Cebpb* was introduced into the cells using Entranster-H4000 transfection reagent (Engreen). After transfection for 24 h, the culture medium was changed, and the cells were further cultured for an additional 24 h. Finally, cell culture supernatants were collected and stored at -80 °C. The siRNA sequences used were as follows: SS sequence: CUGAGCGACGAGUACAAGA; AS sequence: UUGUACUCGUCGCUCAGCU.

### CUT-RUN experiment

Rabbit anti-Cebpb (Genetex, GTX15050) and normal rabbit IgG (Millipore, PP648) were used in this experiment, with 3 × 10^5 freshly separated primary TM used for each experimental group. The experiment was conducted according to the recommended method of the Hyperactive pG-MNase CUT&RUN Assay Kit for PCR/qPCR (Vazyme Biotech, HD101). The amplified DNA fragments were subjected to gel electrophoresis in 3% agarose. The primer sequences are shown in Table 1; The primers for Spike in DNA are provided by the aforementioned kit.

### Statistics

Statistical analysis was performed using Student’s *t-test*, one-way ANOVA followed by Dunnett’s or Tukey’s multiple comparison tests as appropriate. Significance was set at *p* < 0.05. Unless stated otherwise, values are presented as means ± SEM (*indicates *p*<0.05, **indicates *p*<0.01, ***indicates *p*<0.001, ****indicates *p*<0.0001, ns indicates no significance).

## Result

### Transcriptomics reveals the involvement of TM in de novo steroid synthesis

**Fig. 1:**
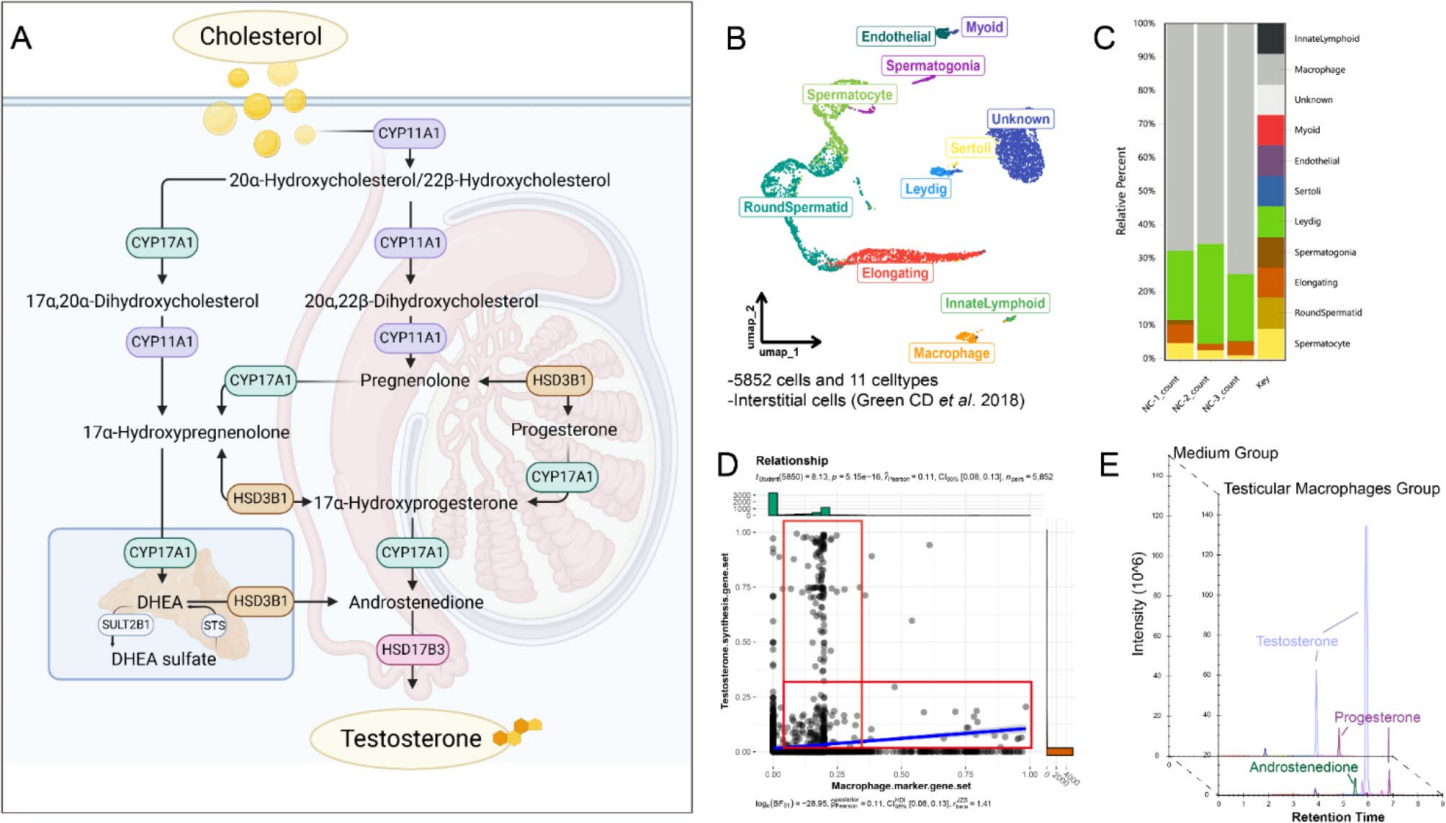
TM and Testosterone Synthesis Genes. **A**: A schematic representation of the complete pathway for de novo testosterone synthesis; **B**: A UMAP plot showing the distribution of different cell clusters in the testis, with each colored dot representing a cell; **C**: The estimated proportions of various cell types following deconvolution analysis of transcriptomic sequencing from three groups of TM; **D**: A scatter plot illustrating the correlation analysis results after scoring each cell for two gene sets; **E**: The results from targeted steroid hormone metabolomics revealed the levels of various steroid hormones in macrophage culture medium and in control blank culture medium

We successfully isolated TM from wild-type mice using F4/80 magnetic beads and subsequently performed transcriptomic sequencing on the harvested cells. The sequencing data revealed a marked expression of genes crucial for de novo testosterone synthesis, specifically *Cyp11a1*, *Cyp17a1*, *Hsd3b1*, and *Hsd17b3* (Table 2) [30]. Typically, Cyp11a1 catalyzes the conversion of cholesterol into pregnenolone, which is then transformed into progesterone by Hsd3b1. This is followed by Cyp17a1 converting progesterone into androstenedione, and ultimately, Hsd17b3 synthesizes testosterone. This process mainly occurs in the Leydig cells of testis, and DHEA secreted by the adrenal glands can also participate in it (Fig. 1A).

**Table 2:** Counts values of partial genes in 3 testicular macropage samples

gene_name	Cyp11a1	Cyp17a1	Hsd3b1	Hsd17b3
NC-1_count	4420.28	21632.9	4658.33	2652.68
NC-2_count	5737.13	27157.2	6953.69	2258.94
NC-3_count	5555.44	19088.9	4701.26	1629.81

*Note* Counts refer to the number of sequencing reads aligned to each gene, indicating the level of gene expression. Higher counts suggest higher gene expression levels

Employing the mouse testicular Drop-seq dataset by Green CD et al., in conjunction with CIBERSORTx for the deconvolution analysis of our sequencing data [31], we ascertained that approximately 70% of the cells identified in the transcriptomic sequencing were TM, while roughly 20% were Leydig cells (Fig. 1B, C). Consequently, this data might not comprehensively represent the authentic expression profile of these four genes specifically in macrophages.

Single-cell sequencing allows for higher-resolution detection of gene expression in tissue cells. However, the low sequencing depth characteristic of Drop-seq limited our ability to interpret the results accurately [32]. Alternatively, we used *Cyp11a1*, *Cyp17a1*, *Hsd3b1*, and *Hsd17b3* as the Testosterone Synthesis Gene Set and *Apoe*, *Adgre1*, *Cd68*, *Lyz2*, and *C1qa* as the Macrophage Marker Gene Set. We conducted a correlation analysis between these two sets of genes to macroscopically examine the expression of testosterone synthesis genes in macrophages. The results suggest that a considerable proportion of macrophages can express the series of genes involved in testosterone synthesis (Fig. 1D). Additionally, we detected an extremely high concentration of testosterone in the culture medium of F4/80-positive TM (Fig. 1E).

### Macrophages can autonomously synthesize and secrete testosterone

**Fig. 2:**
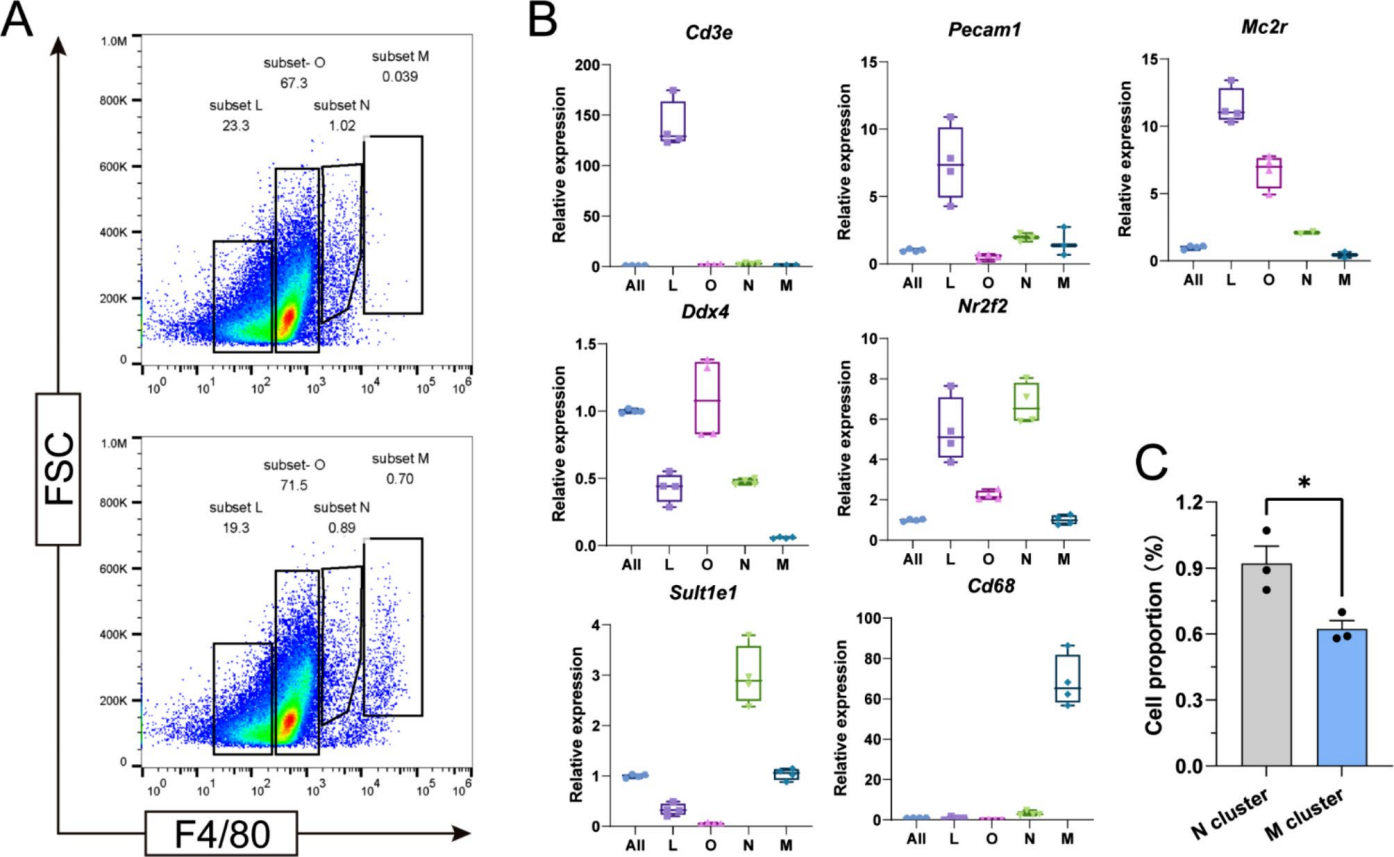
Sorting and Identification of TM and Adult Leydig Cells. **A**: Flow cytometry results of testicular interstitial cells following F4/80–488 staining; **B**: Quantitative PCR results for various marker genes in each cell group

FACS is a highly sophisticated technique for purifying cell populations of interest, achieving a very high purity (95–100%) of the sorted population [33]. To investigate the macrophages within the testis, we collected interstitial cells from the testis of normal wild-type mice and stained them with an Alexa Fluor^®^ 488 conjugated anti-mouse F4/80 antibody. Through the application of FACS techniques, we discovered that under the 488 nm wavelength laser channel, cells were divided into four groups, designated as L, O, N, and M groups (Fig. 2A). As reported, normal interstitial cells produce spontaneous fluorescence under 488 nm laser excitation, forming a separate group, namely Group N, while Groups L and O primarily consist of interstitial stem cells and germ cells, respectively [34]. Based on the marker genes of various testicular cells, we performed qPCR validation for cells in each group [35–37]. The results indicated that Group L contains a rich presence of T cells (*Cd3e*), endothelial cells (*Pecam1*), FLC (*Mc2r*), and Leydig stem cells (*Nr2f2*). Group O comprised FLC (*Mc2r*), germ cells (*Ddx4*), and Leydig stem cells (*Nr2f2*). Group N predominantly consists of ALC (*Sult1e1*), while Group M mainly contained TM (*Cd68*) (Fig. 2B). The macrophage content in Group M reached approximately 98% (Fig.S1). Among these, the ratio of the Group N to the Group M is approximately 3:2 (Fig. 2C).

**Fig. 3:**
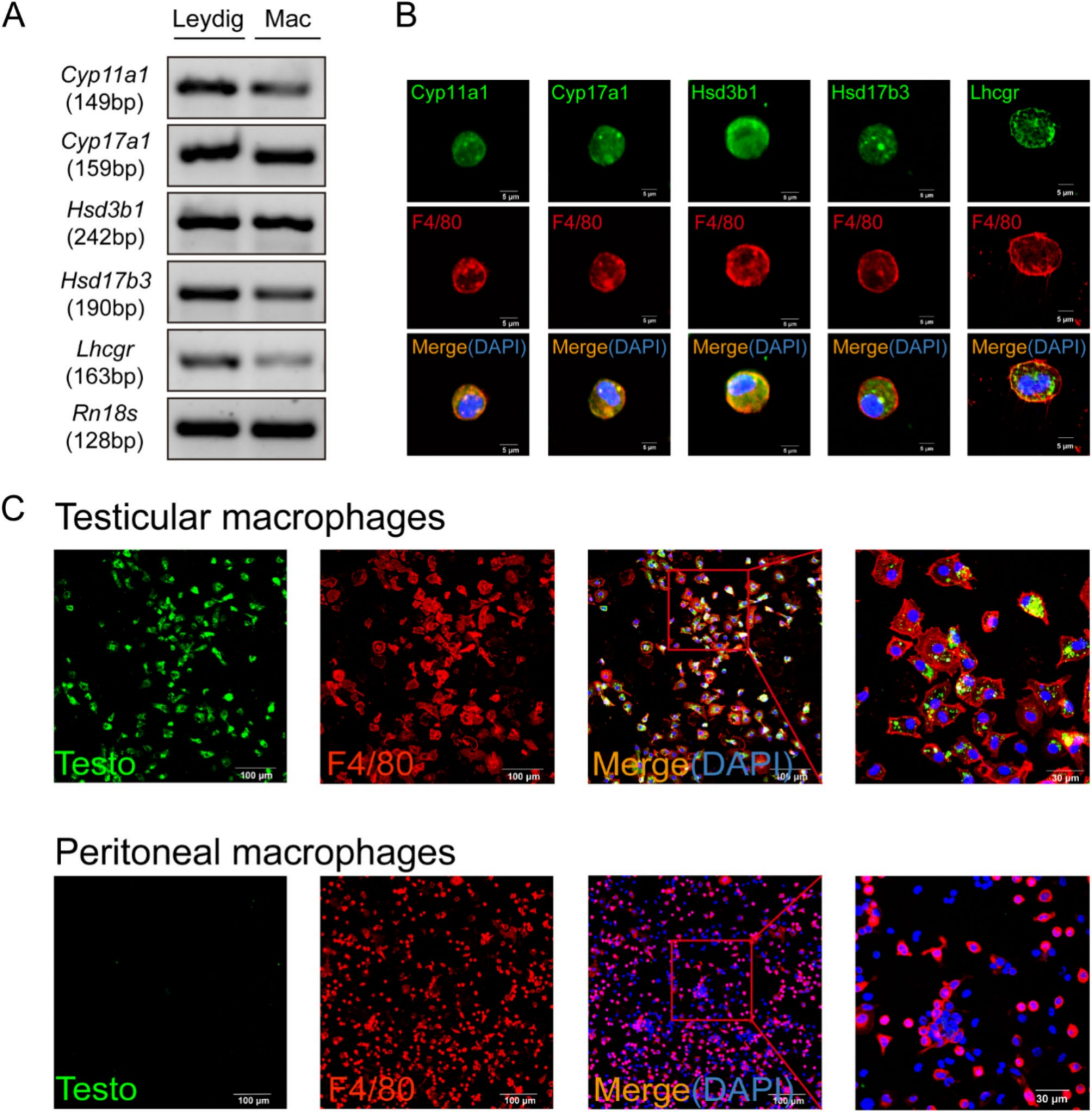
Expression of Testosterone Synthesis Genes in TM. **A**: Comparison of mRNA expression levels of key genes and Lhcgr involved in testosterone synthesis in Adult Leydig cells and TM; **B**: Immunofluorescence images of the four key enzymes, Lhcgr, and the macrophage marker protein F4/80 in mouse TM. Scale bars, 5 μm; **C**: Immunofluorescence images of testosterone and F4/80 in mouse TM and peritoneal macrophages. Scale bars, 100 μm

We analyzed the mRNA levels of four genes and *Lhcgr* involved in testosterone synthesis in ALC (N group) and TM (M group). The results indicated significant expression of these genes in both cell types, but with higher expression levels in Leydig cells. Among these, *Hsd17b3* and *Lhcgr* exhibited weaker expression in TM (Fig. 3A). At the protein level, immunofluorescence staining revealed that proteins Cyp11a1, Cyp17a1, Hsd3b1, Hsd17b3 and Lhcgr are all expressed in F4/80 positive cells (macrophages) (Fig. 3B). Furthermore, we used a testosterone hormone-specific fluorescent antibody for direct immunostaining of primary macrophages. This revealed a substantial presence of testosterone in the cytoplasm of TM, whereas testosterone was not detectable in peritoneal macrophages (Fig. 3C).

**Fig. 4:**
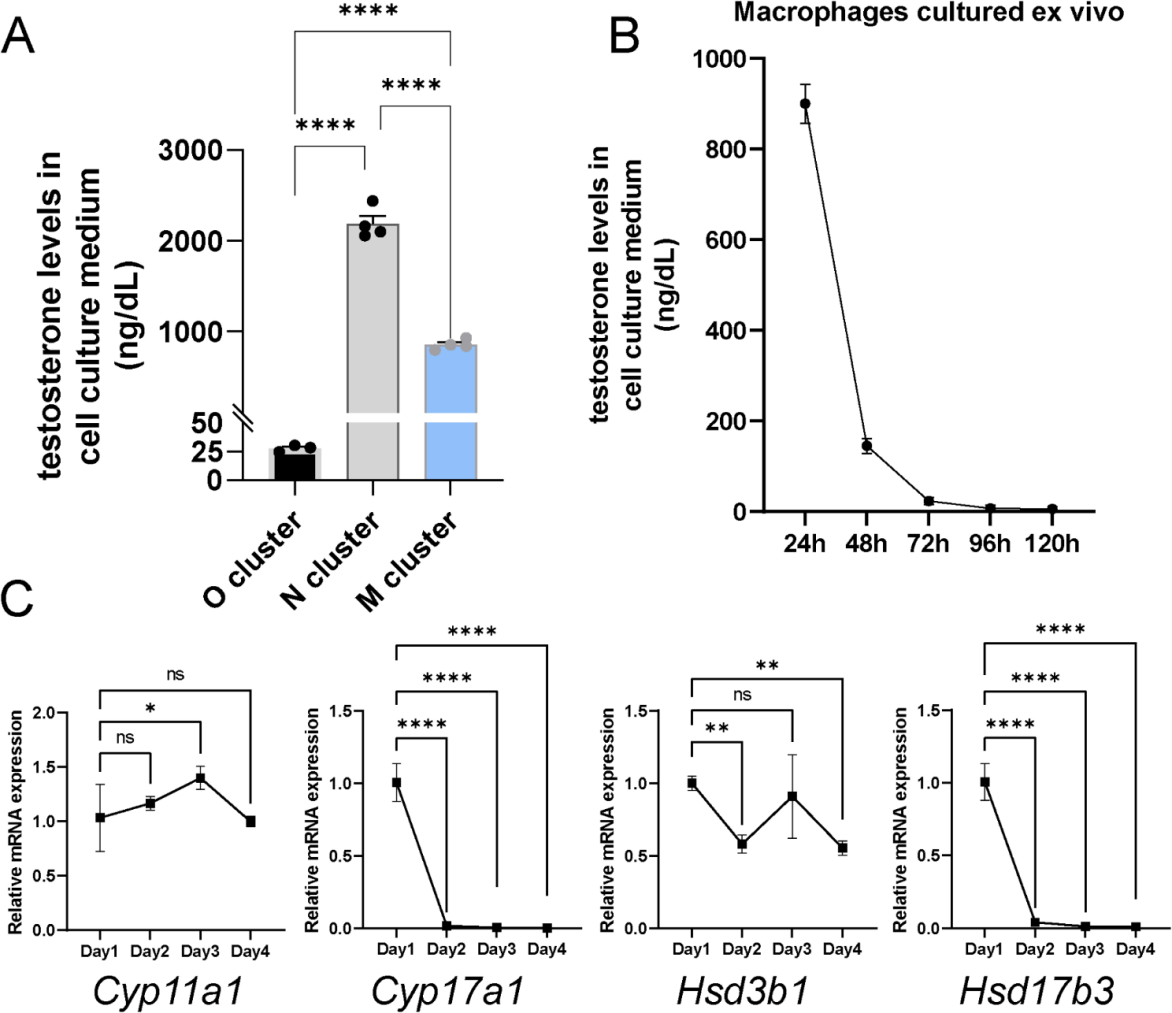
Quantification and Tracking of Testicular Macrophage Secretory Capability. **A**: Testosterone secretion levels in equal O, N, and M cell groups; **B**: Time-dependent changes in testosterone secretion by TM in vitro culture; **C**: Changes in mRNA levels of four key genes in macrophages at different culture times

Equal numbers of cells from groups O, N, and M were cultured in vitro for 24 h. We observed that the O group possessed only limited secretory capabilities, whereas TM from the M group were able to secrete testosterone at levels approximately one-third of those produced by the N group (ALC) (Fig. 4A). However, this secretory ability significantly decreased within 72 h and eventually ceased entirely (Fig. 4B). By tracking the expression changes of four key genes, we discovered that this loss of function was associated with the cessation of *Cyp17a1* and *Hsd17b3* expression (Fig. 4C).

### Depletion of TM affects physiological testosterone levels in mice

**Fig. 5:**
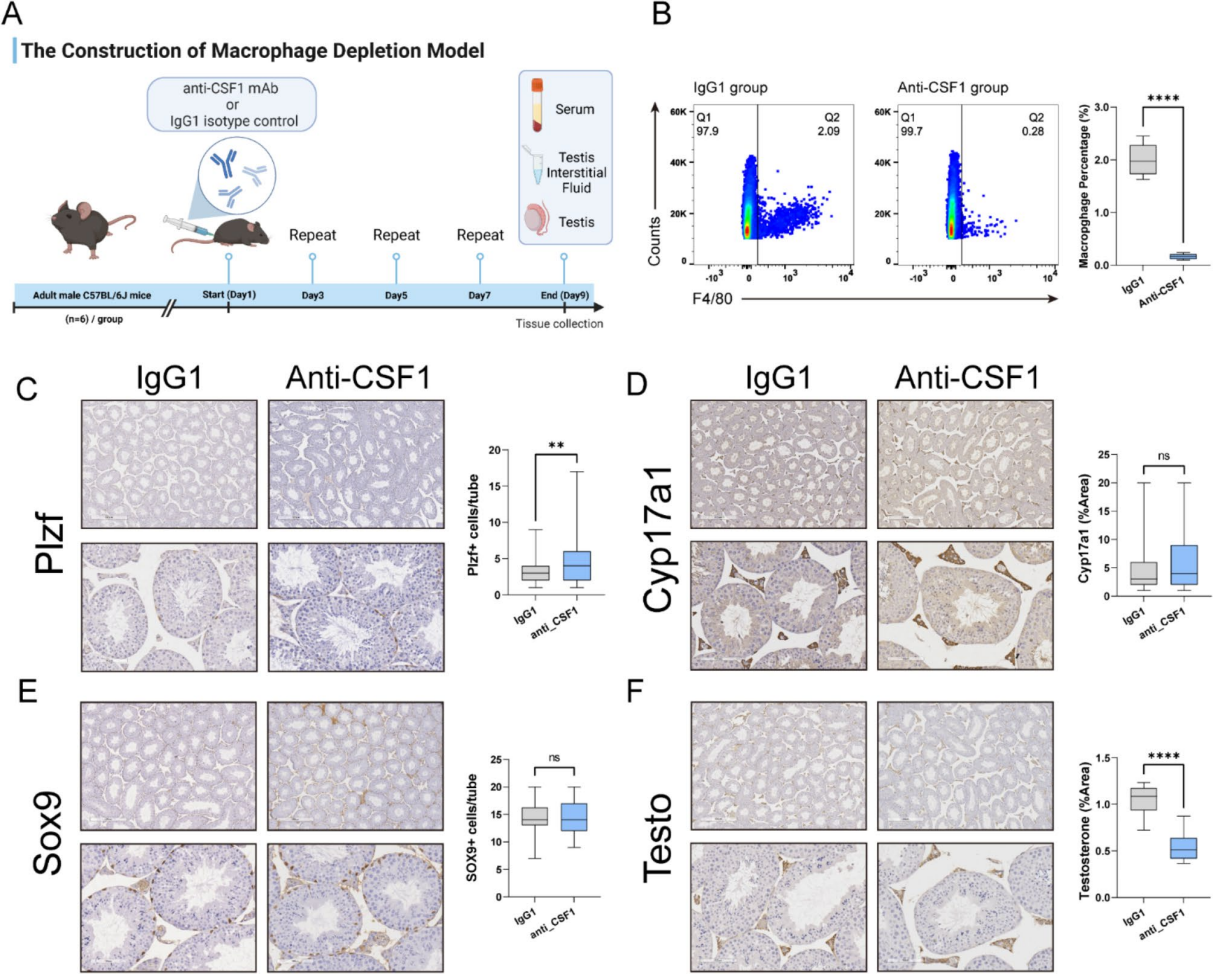
Construction and Validation of Macrophage Depletion Mouse Model. **A**: Schematic diagram of the construction of the testicular macrophage depletion model in mice; **B**: Flow cytometry validation of the proportion of F4/80 positive cells in the total testis of macrophage-depleted mice compared to control mice; **C**, **D**, **E**, **F**: Immunohistochemical analysis showing changes in spermatogonial stem cells (Plzf), Interstitial cells (Cyp17a1), Sertoli cells (Sox9), and Testosterone after macrophage depletion, with bar graphs summarizing the results of the immunohistochemical analysis

To more directly evaluate the significance of autonomous testosterone production and secretion by TM, we ablated macrophages within the mouse testis using a CSF1 neutralizing antibody (Fig. 5A). Flow cytometry was used to verify the depletion efficiency: compared to the control group, the proportion of macrophages in the experimental group decreased from 2% to approximately 0.2%, indicating successful macrophage depletion (Fig. 5B). To assess whether this treatment affected other cells in the testicular tissue, we performed immunohistochemical staining for various cell marker proteins. The results demonstrated a modest increase in the number of spermatogonial stem cells (Plzf) in the short term (*p* < 0.01). In contrast, no statistically significant differences were observed in the counts of interstitial cells (Cyp17a1) and Sertoli cells (Sox9) before and after the treatment (*p* > 0.05) (Fig. 5C, D, E).

**Fig. 6:**
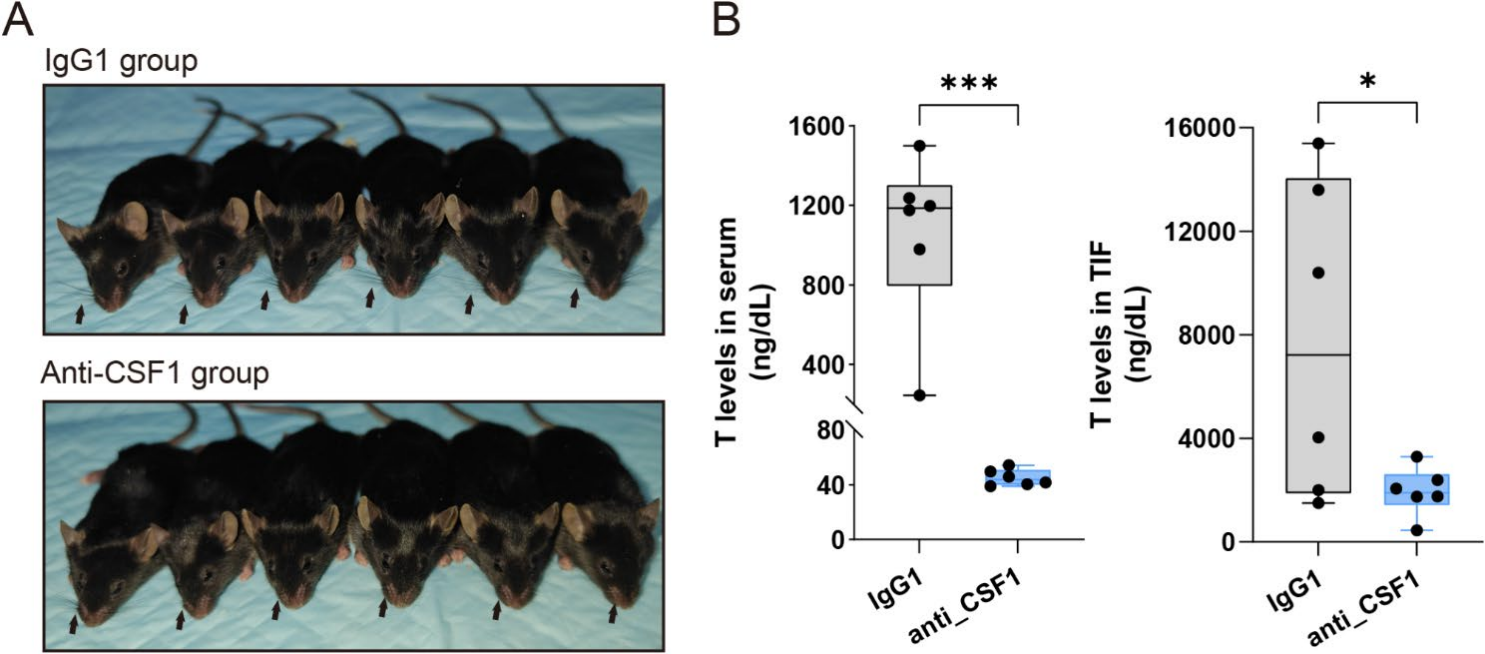
Whiskers and Steroid Hormone Levels in Macrophage Depletion Mice. **A**: Whiskers of control group and macrophage depletion mice, indicated by arrows; **B**: Changes in testosterone levels in serum and testicular interstitial fluid

In mice, the reduction of macrophages in the testis led to a notable phenotype characterized by the loss of whiskers (Fig. 6A). Additionally, there was a significant decrease in testosterone levels in both serum and TIF (*p* < 0.05) (Fig. 6B). Immunohistochemical examination of the testicular tissues showed that the staining intensity of testosterone was reduced by approximately 50% (Fig. 5F).

### Cebpb regulates macrophage testosterone secretion

**Fig. 7:**
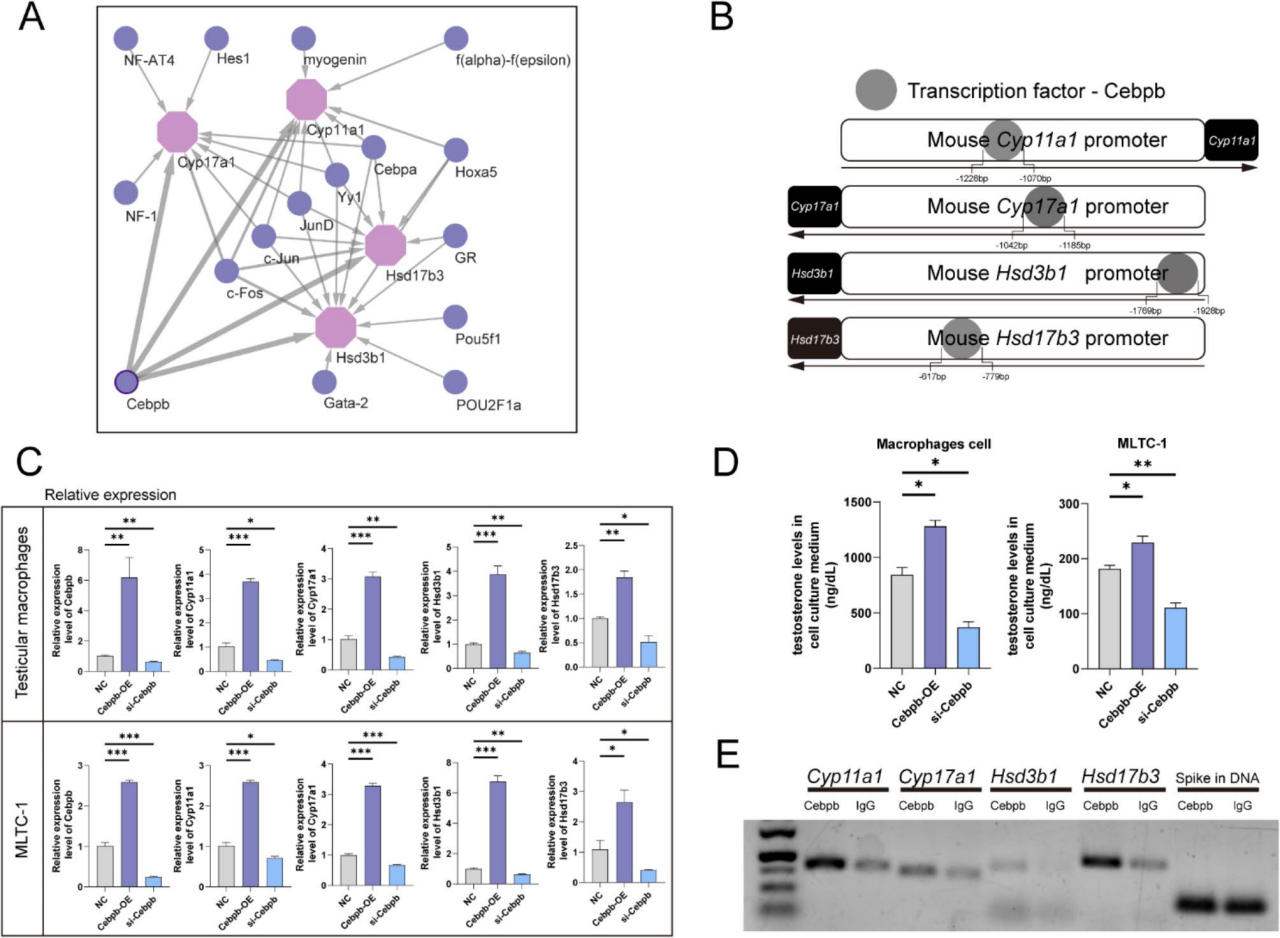
Cebpb is an upstream regulator of testosterone secretion in TM. **A**: Network diagram showing all transcription factors predicted based on the four target genes, with the thickness of the arrows corresponding to the RE values; **B**: A schematic illustrating the binding sites of Cebpb with various gene promoter regions; **C**: Bar graphs showing the changes in relative mRNA expression levels of different genes in cells with overexpression and knockdown of Cebpb; **D**: Bar graphs depicting the variations in testosterone hormone secretion in two groups of cells following overexpression and knockdown of Cebpb; **E**: Agarose gel electrophoresis displaying the content of different gene 5` regulatory region fragments, with Spike in DNA serving as an internal control to ensure equal total DNA content in both groups

We used PROMO to predict the upstream transcription factors of a series of genes involved in testosterone synthesis and identified Cebpb as a potential regulatory factor (Fig. 7A, B). To determine the regulatory role of Cebpb in testosterone synthesis, plasmids overexpressing *Cebpb* and siRNAs targeting *Cebpb* were transfected into primary TM and the Leydig cell line MLTC-1, which also synthesize testosterone. The qPCR experiments showed that the transcription levels of *Cyp11a1*, *Cyp17a1*, *Hsd3b1*, and *Hsd17b3* in both cell types synchronously increased or decreased with the upregulation or downregulation of *Cebpb* (Fig. 7C). Additionally, testosterone levels in the culture medium of TM and MLTC-1 cells significantly increased following *Cebpb* overexpression and decreased after *Cebpb* knockdown (Fig. 7D). CUT-RUN assays demonstrated the binding of Cebpb to the promoters of *Cyp11a1*, *Cyp17a1*, *Hsd3b1*, and *Hsd17b3* in TM (Fig. 7E). Overall, our data suggest that Cebpb can regulate de novo synthesis of testosterone in TM by modulating the expression of these four key genes and can exert similar effects in MLTC-1 cells.

## Discussion

For decades, the potential functions of TM have been extensively studied. They represent the second most abundant cell population in the testicular interstitium, with an average ratio of one macrophage to every eight Leydig cells [38]. Structurally, TM and Leydig cells are coupled through specialized membrane structures composed of microvilli from Leydig cells within the macrophage-coating vesicles, forming an essential structure for Leydig cell function [38]. Functionally, TM secrete 25-hydroxycholesterol, providing substrates for Leydig cell testosterone synthesis [39]. Multiple studies by Gaytan and colleagues have shown that in the absence of TM, Leydig cell proliferation, differentiation, and response to LH/hCG are significantly affected [14, 40, 41]。Cohen and Kmicikiewicz further confirmed that the in vivo depletion of TM reduces testosterone levels, while co-culturing Leydig cells with TM or directly using testicular macrophage-conditioned medium (TMCM) can significantly increase testosterone production [42–46]. Therefore, based on existing reports, it can be confirmed that TM play an important role in testosterone hormone synthesis. Research on the Leydig cell ablation model indicates that even after the loss of Leydig cells, the testes can still maintain a certain level of testosterone synthesis. This suggesting that other cells within the testes can produce testosterone [47, 48].Our study directly demonstrates that TM have the capacity to synthesize testosterone by detecting the expression of the testosterone synthesis pathway in TM.

Compared to traditional RNA sequencing (RNA-seq), single-cell RNA sequencing (scRNA-seq) offers a more accurate and extensive approach for depicting changes and mechanisms at the cellular level [49]. scRNA-seq allows us to explore gene expression in tissue cells with higher resolution. In single-cell studies of the testis, the expression of *Cyp11a1*, *Cyp17a1*, *Hsd3b1*, and *Hsd17b3* is typically confined to Leydig cells rather than TM [50–53]. However, some researchers, based on the adhesion characteristics of different cells, have isolated TM and discovered their innate ability to synthesize progesterone and express *Cyp11a1* normally, consistent with the findings of our study [18]. This discrepancy between single-cell sequencing results and actual gene expression may be attributed to the inherent sensitivity limitations of single-cell sequencing, which may not fully capture the complete gene expression profile of macrophages [54].

To accurately investigate whether TM can autonomously synthesize testosterone de novo, a comprehensive validation from the genetic to the functional level is necessary. We discovered that TM are capable of expressing the key enzymes required for de novo synthesis of testosterone: Cyp11a1, Cyp17a1, Hsd3b1, Hsd17b3, and related receptors Lhcgr. Moreover, extremely high levels of testosterone were detectable both in the culture medium of TM and within the cytoplasm. Our findings suggest that the ratio of ALC to TM is approximately 3:2, with their secretory capacities in a 2:1 ratio under equal cell conditions, indicating that TM contribute significantly to testicular testosterone levels. Furthermore, we observed that TM lose the expression of *Cyp17a1* and *Hsd17b3* and the ability to secrete testosterone within 72 h of in vitro culture. A similar phenomenon is observed in other testicular cells. For instance, Pdgfr-α positive peritubular myoid cells gradually lose the expression of *Cyp11*, *Hsd3b1*, and *Cyp17* within 7 days of culture, while rat ALC progressively lose the capability to synthesize testosterone within 5 days of in vitro culture [55, 56]. In the study by Gary R. Klinefelter et al., the amount of testosterone production by testicular Leydig cells decreases to 30–40% of the control group after 4 days of in vitro culture, with Cyp17a1 enzyme content at 39% of the control group; after 12 days, the enzyme content is only 12% of the control group [57]. This suggests that both testicular macrophages and Leydig cells cannot maintain long-term testosterone synthesis functions in vitro, indicating significant differences between in vitro and in vivo environments.

Similar to TM, Bidesh Mahata et al. discovered that T cells can synthesize pregnenolone de novo [58]. Milewich L et al. found that human alveolar macrophages can convert androstenedione to testosterone and other steroids through the catalytic activities of enzymes like 3β-HSD, 3α-HSD, 17β-HSD, and 5α-reductase. These steroidogenic enzymes are also present in porcine alveolar macrophages [59, 60]. Similarly, testosterone is converted into androstenedione and dihydrotestosterone in primary cultured human synovial macrophages [10, 61]. Additionally, in the presence of LPS, human monocyte-derived macrophages (but not monocytes) preferentially convert dehydroepiandrosterone into physiologically relevant amounts of downstream steroid hormones, including testosterone, androstenedione, estrone, and estradiol [62, 63]. These results collectively suggest that macrophages have the capability to generate and transform steroid hormones, and their functional roles may vary depending on the microenvironment and their state of differentiation.

CSF1 is a crucial cytokine that regulates the proliferation, differentiation, maturation, and survival of the mononuclear phagocyte system. Continuous CSF1R signaling is essential for maintaining the macrophage population in adult mice [64–67]. Blocking CSF1 action is a well-established method for depleting resident tissue macrophages in various organs. In our study, CSF1 neutralizing antibodies were used to reduce TM by approximately 90%, while avoiding effects on the number of Sertoli cells and Leydig cells. The unchanged area of Cyp17a1-positive cells is attributed to the fact that TM only constitute 1/9 of the interstitial cells, making their reduction unlikely to significantly affect the overall levels of the positive cell population. The short-term increase in spermatogonial stem cells may be related to the regulatory role of CSF1 and changes in the functional activities of macrophages [42, 66]. Moreover, following macrophage depletion, we observed a dramatic decline in testosterone levels both within the testis and in serum, which was accompanied by noticeable whisker loss in mice, closely related to the decrease in testosterone levels [68]. These findings underscore the critical role of TM in testosterone synthesis within the testis.

Transcription factors play a crucial role in regulating gene expression by binding to the promoter regions of target genes [69–71]. Through bioinformatics predictions, we identified potential binding sites for Cebpb in the promoter regions of *Cyp11a1*, *Cyp17a1*, *Hsd3b1*, and *Hsd17b3*. In this study, supported by bioinformatic analysis, qPCR, testosterone hormone secretion assays, and CUT-RUN, we have established that Cebpb can regulate the expression of these four genes within the testosterone synthesis pathway. Crucially, we verified that Cebpb directly influences testosterone secretion levels. Thus, Cebpb positively modulates the synthesis and secretion of testosterone in TM by regulating the transcription of genes involved in the testosterone synthesis pathway.

In summary, we have confirmed a groundbreaking discovery that TM are capable of secreting testosterone. Traditionally, TM were primarily considered effector cells in immune responses, responsible for clearing viruses, bacteria, and damaged cells [72]. Conversely, testosterone production was primarily attributed to Leydig cells. The revelation that macrophages can also produce testosterone indicates that their functions in the reproductive and endocrine systems extend beyond immune regulation. This discovery enhances our comprehension of hormonal regulation, suggesting that other cells, besides the well-established primary hormone-producing cells, can also influence hormone levels. It unveils a more intricate endocrine regulatory network within the human body. If the testosterone production by macrophages is related to certain diseases or physiological conditions, such as male infertility or other hormonal imbalances, targeting macrophages for treatment could become a new therapeutic strategy. Moreover, the development of new drugs targeting testosterone production by macrophages or their interactions with other hormones or signaling pathways could offer novel treatment strategies for related diseases. In summary, the ability of TM to secrete testosterone will redefine their roles in reproductive physiology and may open up new avenues for research and treatment.

## Conclusions

This study demonstrates that TM can independently synthesize and secrete testosterone, significantly contributing to testosterone levels within the testes. This research highlights the critical role of the transcription factor Cebpb in regulating the expression of genes related to testosterone synthesis in macrophages, unveiling the potential regulatory mechanism of Cebpb in this process.

## Electronic supplementary material

Below is the link to the electronic supplementary material.

Additional file 1: Figure S1. Analysis of Surface Markers in F4/80 Positive Cell Populations. A: Flow cytometry results showing the positivity rates of CD45, CD11b, and CD68 in F4/80 positive cell populations. B: Bar graph summarizing the proportions of CD45, CD11b, and CD68 positive cells within the F4/80 positive cell population

## Data Availability

The raw RNA sequence data reported in this paper were deposited at the National Genomics Data Center (www.ngdc.cncb.ac.cn), under the accession number CRA013364. The codes supporting the current study are available from the corresponding author on reasonable request.
