# Supplementary material for: Mouse testicular macrophages can independently produce testosterone and are regulated by Cebpb
Source: Biol Res. 2024 Sep 9;57:64. doi: 10.1186/s40659-024-00544-8 (PMC11382419; doi:10.1186/s40659-024-00544-8)

Figure S1. Analysis of Surface Markers in F4/80 Positive Cell Populations. A: Flow cytometry results showing the positivity rates of CD45, CD11b, and CD68 in F4/80 positive cell populations. B: Bar graph summarizing the proportions of CD45, CD11b, and CD68 positive cells within the F4/80 positive cell population.


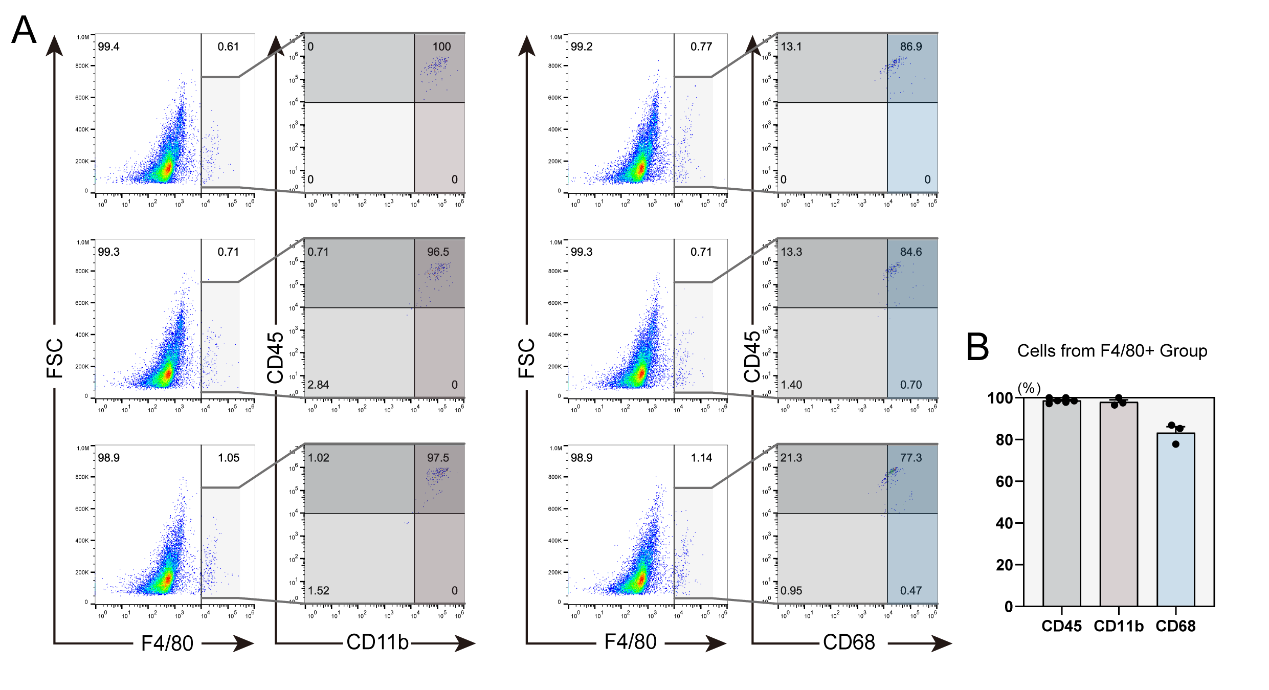

Supplement: Supplementary file 1 — Additional file 1: Figure S1. Analysis of Surface Markers in F4/80 Positive Cell Populations. A: Flow cytometry results showing the positivity rates of CD45, CD11b, and CD68 in F4/80 positive cell populations. B: Bar graph summarizing the proportions of CD45, CD11b, and CD68 positive cells within the F4/80 positive cell population [file 40659_2024_544_MOESM1_ESM.docx]
